# Development and validation of deep learning models for identifying the brand of pedicle screws on plain spine radiographs

**DOI:** 10.1002/jsp2.70001

**Published:** 2024-09-17

**Authors:** Yu‐Cheng Yao, Cheng‐Li Lin, Hung‐Hsun Chen, Hsi‐Hsien Lin, Wei Hsiung, Shih‐Tien Wang, Ying‐Chou Sun, Yu‐Hsuan Tang, Po‐Hsin Chou

**Affiliations:** ^1^ School of Medicine National Yang Ming Chiao Tung University Taipei Taiwan; ^2^ Department of Orthopedics and Traumatology Taipei Veterans General Hospital Taipei Taiwan; ^3^ Department of Orthopaedic Surgery, National Cheng Kung University Hospital, College of Medicine National Cheung Kung University Tainan Taiwan; ^4^ Program of Artificial Intelligence and Information Security Fu Jen Catholic University New Taipei City Taiwan; ^5^ Department of Orthopedics Shin Kong Wu Ho‐Su Memorial Hospital Taipei Taiwan; ^6^ Kinmen Hospital Ministry of Health and Welfare Kinmen Taiwan; ^7^ Department of Radiology Taipei Veterans General Hospital Taipei Taiwan; ^8^ Department of Medical Imaging and Radiological Technology Yuanpei University of Medical Technology Hsinchu Taiwan

**Keywords:** artificial intelligence, deep learning, instrumentation, pedicle screws

## Abstract

**Background:**

In spinal revision surgery, previous pedicle screws (PS) may need to be replaced with new implants. Failure to accurately identify the brand of PS‐based instrumentation preoperatively may increase the risk of perioperative complications. This study aimed to develop and validate an optimal deep learning (DL) model to identify the brand of PS‐based instrumentation on plain radiographs of spine (PRS) using anteroposterior (AP) and lateral images.

**Methods:**

A total of 529 patients who received PS‐based instrumentation from seven manufacturers were enrolled in this retrospective study. The postoperative PRS were gathered as ground truths. The training, validation, and testing datasets contained 338, 85, and 106 patients, respectively. YOLOv5 was used to crop out the screws' trajectory, and the EfficientNet‐b0 model was used to develop single models (AP, Lateral, Merge, and Concatenated) based on the different PRS images. The ensemble models were different combinations of the single models. Primary outcomes were the models' performance in accuracy, sensitivity, precision, F1‐score, kappa value, and area under the curve (AUC). Secondary outcomes were the relative performance of models versus human readers and external validation of the DL models.

**Results:**

The Lateral model had the most stable performance among single models. The discriminative performance was improved by the ensemble method. The AP + Lateral ensemble model had the most stable performance, with an accuracy of 0.9434, F1 score of 0.9388, and AUC of 0.9834. The performance of the ensemble models was comparable to that of experienced orthopedic surgeons and superior to that of inexperienced orthopedic surgeons. External validation revealed that the Lat + Concat ensemble model had the best accuracy (0.9412).

**Conclusion:**

The DL models demonstrated stable performance in identifying the brand of PS‐based instrumentation based on AP and/or lateral images of PRS, which may assist orthopedic spine surgeons in preoperative revision planning in clinical practice.

## Graphical abstract

The AP + Lateral ensemble model had the most stable performance in terms of accuracy, F1 score, and AUC. The performances of the ensemble models were comparable to that of experienced orthopedic spine surgeons. The external validation also confirmed the good performance of DL models reported in this study.

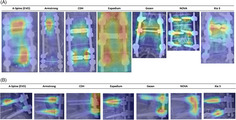

## 1 INTRODUCTION

Pedicle screw (PS)‐based instrumentation is the commonly used internal fixation device for the treatment of spinal degenerative disease, deformities, tumors, and fractures.^1^ However, symptomatic adjacent segment degeneration^2^ and failed back surgery syndrome^3^ are common reasons for revision surgery. In spinal revision surgery, previous implants may need to be removed and replaced with new implants. Hence, orthopedic surgeons must accurately identify the brand of the existing implants and gather the appropriate surgical equipment for implant removal, since the universal removal set is expensive and may not be available in all hospitals. Failure to accurately identify PS‐based instrumentations preoperatively may increase the surgical time and the risk of perioperative complications.

In clinical practice, implants are typically identified using plain radiographs of the spine (PRS). Deep learning (DL) models have been applied to identify fractures on plain radiographs with expert‐level accuracy.^4^, ^5^, ^6^, ^7^ Additionally, numerous studies suggested the potential of DL models to recognize knee and hip arthroplasties,^8^, ^9^, ^10^ and cervical plating systems.^11^, ^12^ Yang et al.^13^ reported that a variety of DL models are effective for one‐segment spinal implant identification, yielding 76.0%–98.7% precision and 72.0%–98.4% recall; however, the performance of DL models in identifying spinal implants in multi‐segment fixation has not been investigated yet. While DL models have been used to identify the shaft of PS in the PRS^14^ and the surrounding pedicle anatomy in CT scans,^15^ these studies did not address the ability of DL models to identify the device manufacturer. Moreover, the generalizability of the ground truth plays an important role in the performance of DL models.^7^

We hypothesized that the DL model may have stable performance in identifying PS‐based instrumentation in the PRS and that the ground truth of different images on the PRS may affect the DL model performance. The objectives of this study were as follows: (1) to develop various DL models based on the different ground truths of PRS on anteroposterior (AP) and lateral images and to evaluate their performance in identifying different brands of PS‐based instrumentation; (2) to investigate the effect of PRS at AP or lateral images on the performance of the DL models; (3) to determine whether ensemble methods improve the model's performance and validate the optimal model; and (4) to compare the performance of our models with human readers and assess the performance of the DL models via external validation.

## 2 MATERIALS AND METHODS

### 2.1 Enrolled dataset

This study protocol was approved by the Institutional Review Board of our institution (2022‐05‐007AC). The medical records of patients receiving PS‐based instrumentation surgery from January 1, 2018, to June 30, 2020, at our institution were retrospectively reviewed. The exclusion criteria included mismatched brands between instrumentation and crosslinks (*n* = 25) and the presence of two brands of instrumentation in one PRS (*n* = 13). The corresponding postoperative PRS on AP and lateral images and the different brands of inserted implants were gathered as our ground truths. A total of 529 patients were included for the development of our DL models.

**FIGURE 1:**
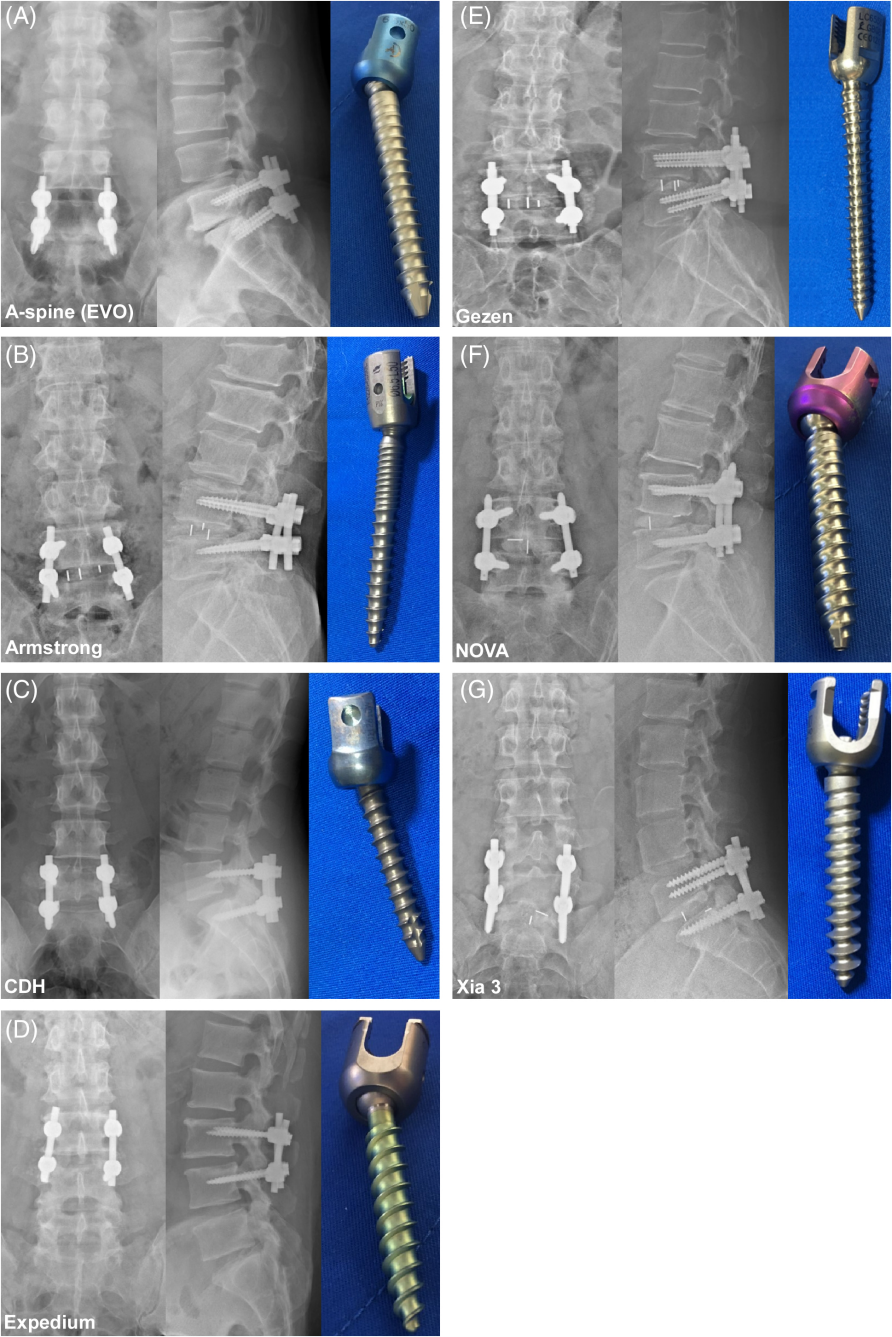
Illustration of the seven enrolled pedicle screw‐based instrumentations on plain radiographs of the spine in anteroposterior (AP) (left) and lateral images (middle), and the whole construct of the screw with head, neck, and body (right). (A) A‐spine (EVO), (B) Armstrong, (C) CDH, (D) Expedium, (E) Gezen, (F) NOVA, (G) Xia 3.

Seven types of PS‐based instrumentation commonly used in our clinical institution were considered as ground truths, including (1) A‐spine SmartLoc Evolution (EVO) (Smartlock Omega; A‐Spine Inc., New Taipei City, Taiwan), (2) Armstrong (Paonan Biotech [BIOMECH], Taipei City, Taiwan), (3) Gezen (BioLife Medical Device Inc, Hsinchu City, Taiwan), (4) CDH (CDM8; Medtronic, Minneapolis, MN, USA), (5) Expedium (DePuy Synthes Inc., West Chester, PA, USA), (6) NOVA (BAUI Biotech Co., Ltd., New Taipei City, Taiwan), and (7) Xia 3 (Stryker Spine, Allendale, NJ, USA) (Figure 1).

### 2.2 Plain radiography technique

The radiography machine used a high‐voltage generator (UD150B‐40; Shimadzu Corp., Kyoto, Japan) with a voltage of 94 kVp and an average current of 56 mAs for 360 ms. Computer software was used to investigate instrumentation on PRS in the AP and lateral projections (Smart Viewer 3.2; Taiwan Electronic Data Processing Corp., Taipei City, Taiwan).

### 2.3 Development of deep learning models

**FIGURE 2:**
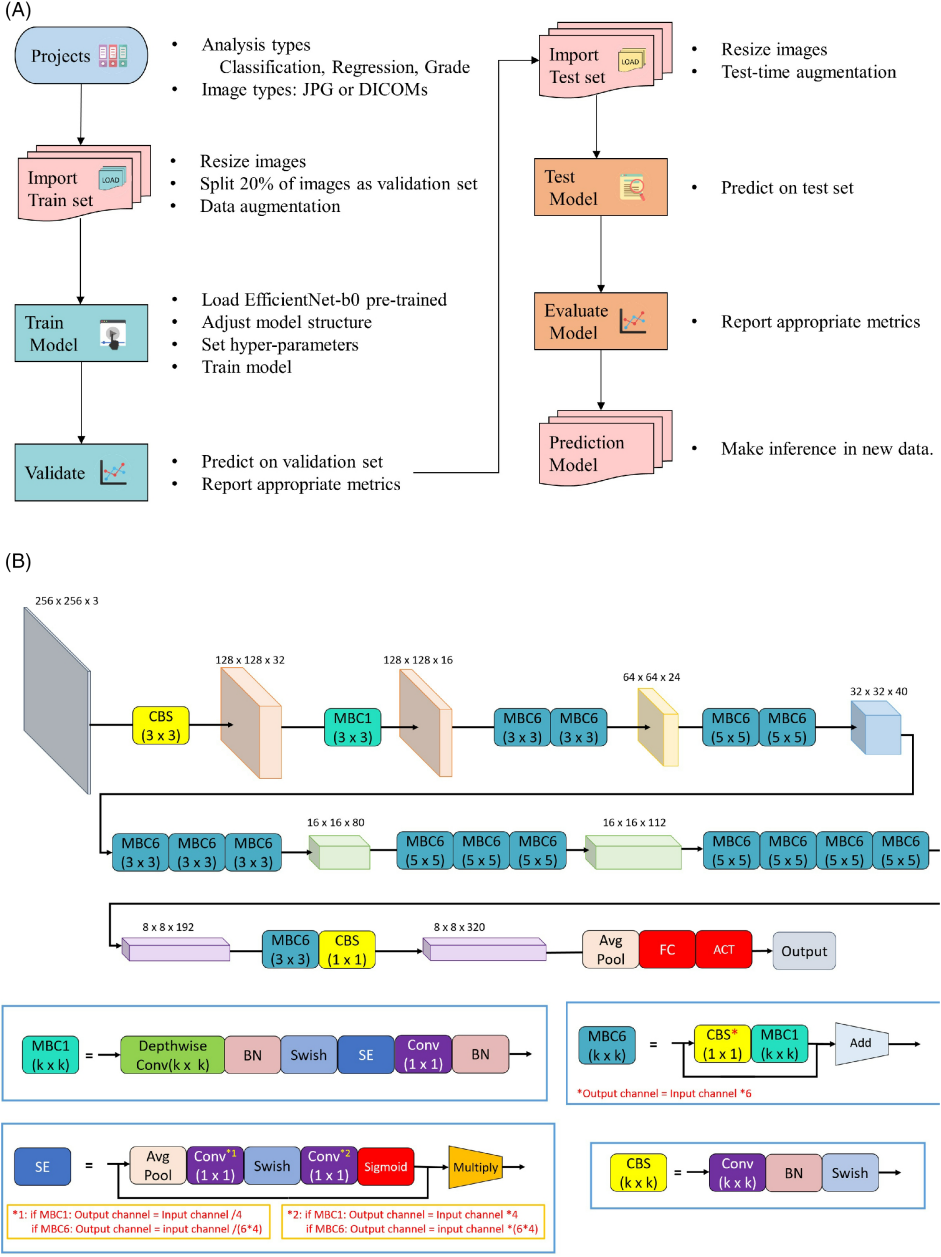
Framework of Medical Artificial Intelligence Aggregator (MAIA) software and the structure of the built‐in EfficientNet‐b0 model. (A) Framework for MAIA software. (B) Structure of the built‐in EfficientNet‐b0 model.

The pre‐trained You Only Look Once version 5 (YOLOv5, arXiv) was used to identify and crop out the trajectory of PS in PRS to enhance the performance of models. Medical Artificial Intelligence Aggregator (MAIA) software (Muen Biomedical and Optoelectronic Technologist, Inc., Taipei City, Taiwan) was used for automated analysis of the medical images based on the structure of the built‐in, pre‐trained EfficientNet‐b0 model on ImageNet (Figure 2).^16^, ^17^ The graphic processing unit was NVIDIA GeForce RTX 2070. Image file formats in Digital Imaging and Communications in Medicine (DICOM) were imported into MAIA, which automatically adjusted the model structure to adapt to the analysis type (i.e., classification, regression, or grading). The images were then resized to 256 × 256 with 3 color channels, and Horizontal Flip and Rotate methods were used for data augmentation to prevent over‐fitting.^18^ The batch size was decided according to the memory consumption. The loss function was calculated by cross‐entropy loss or mean square error, depending on the type of analysis conducted. An Adam optimizer was used to minimize the loss.^19^ The learning rate was tuned using the one‐cycle of cosine annealing strategy.^20^, ^21^

Under the framework of MAIA, the AP model, Lateral (Lat) model, Concatenated (Concat) model, and Merge model were developed based on different ground truths of AP and/or lateral images. In other words, all four models employed the same EfficientNet‐B0 architecture (pre‐trained on ImageNet) but were fine‐tuned on different image datasets. The AP model was fine‐tuned on the AP images of PRS, and the Lat model was fine‐tuned on the lateral images of PRS. In addition, the AP and lateral images of the PRS were first combined to form a single concatenated image (shape: 256 × 256 × 3); the Concat model was fine‐tuned on concatenated images of PRS. Finally, both AP and lateral images without concatenation were simultaneously used to fine‐tune the Merge model. Therefore, the Merge model produced three predications based on AP images, lateral images, and dual images (both AP and lateral images). The ensemble models were constructed using logistic regression, assembling the predicted probabilities from different combinations of single models.

### 2.4 Datasets for training, validating, and testing

The images of the 529 patients were divided into three groups: training dataset (*n* = 338), testing dataset (*n* = 106), and validation dataset (*n* = 85). The patient groups were stratified by brand and presence of crosslink, which ensured similarity in the ratios of different brands and in the presence of a crosslink in the training, validation, and testing datasets (Table 1). Only the training dataset was used to calculate the gradients and update the model parameters. The validation dataset was used to evaluate the model during each phase of the training process, and the model with the lowest validation loss was selected. Finally, the selected model was evaluated using the testing dataset, which was kept completely independent from the training process.

**TABLE 1:** Number of enrolled patients in training, validation, and test sets according to brand and the presence of cross‐links.

Spinal pedicle	Enrolled patients					
Screw systems	Number of patients			Training	Validation	Test
A‐Spine (EVO)	87	With CL	44	28	6	10
		Without CL	43	27	8	8
Armstrong	85	With CL	48	32	7	9
		Without CL	37	22	7	8
CDH	84	With CL	63	38	12	13
		Without CL	21	17	1	3
Expedium	43	With CL	5	4	0	1
		Without CL	38	23	7	8
Gezen	85	With CL	30	20	4	6
		Without CL	55	34	10	11
NOVA	63	With CL	0	0	0	0
		Without CL	63	41	10	12
Xia 3	82	With CL	14	10	1	3
		Without CL	68	42	12	14
Total	529	With CL	204	132	30	42
		Without CL	325	206	55	64

Abbreviation: CL, crosslink.

### 2.5 DL model evaluation and statistical analysis

The AP, Lat, and Concat models each provided only one prediction per patient. To evaluate the performance of the Merge model, we calculated the performance metrics based on three image datasets of PRS: The Merge model trained on AP images, Merge model trained on lateral images, and Merge model trained on dual images. Accuracy, precision, sensitivity, F1‐score, interobserver reliability (kappa value), and area under the receiver operating characteristic curve (AUC) were calculated to evaluate the performance of the single and ensemble models. These metrics were calculated as either brand‐based or overall evaluation.

In a brand‐based evaluation, all metrics except accuracy were calculated based on each device type, with one type considered positive and all the others considered negative. In an overall manner, the macro‐average and micro‐average were each calculated. The macro‐average was computed by averaging the values of the brand‐based evaluation. The micro‐average was computed by aggregating the results of all brands to define true positive, false positive, true negative, and false negative, which were used to calculate metrics.

In addition to the numeric metrics mentioned above, MAIA also reported graphic illustration of a confusion matrix, receiver operating characteristic (ROC) curve, and a gradient‐weighted class activation map (Grad‐CAM).^22^ Grad‐CAM was used to evaluate the heatmap for evidence that the model recognized the discriminative features of instrumentations, as indicated by a color transition from blue to red. To evaluate the effect of crosslinks on model performance, numeric metrics based on PRS were calculated separately with or without crosslinks.

### 2.6 Comparison of the performance between human readers and DL models

To compare the performance between our DL models and human readers, the AP and Lat images of PRS of 27 patients not included in our dataset were randomly selected from the included 529 patients using the randomization program.^23^ An accurate illustration of each implant was provided for readers beforehand (Figure 1). The six human readers included one medical student, one orthopedic resident, one spine fellow, one general orthopedic surgeon, and two orthopedic spine surgeons. Moreover, five additional orthopedic surgeons (Readers 7–11) from another medical center were invited to participate in the test using the same datasets.

### 2.7 Evaluation of DL models by external validation

For external validation, we obtained a dataset from another medical institution that used a different plain radiographic technique for external validation; these images were from patients in a population bearing the same seven brands of screws (*n* = 31).

## 3 RESULTS

Of the MAIA models, the Lat model had the most stable performance (Table 2). Of the ensemble models, the AP + Lat ensemble model exhibited the most stable performance (Table 3). The performance of the Ensemble models was superior to that of the MAIA models (Table 4).

**TABLE 2:** Brand‐based evaluation of MAIA models, regardless of the presence of cross‐links.

Metrics	AP model	Lat model	Concat model	Merge model			Average
				Dual images	AP images	Lat images	
Accuracy	0.8113	0.9057	0.8585	0.7972	0.7075	0.8868	0.8278
Kappa value	0.7791	0.8894	0.8343	0.7623	0.6580	0.8671	0.7984
Precision							
A‐Spine (EVO)	0.8667	0.9444	1.0000	0.8333	0.8000	0.8571	0.8836
Armstrong	0.8235	1.0000	0.8667	0.7429	0.6111	0.8824	0.8211
CDH	1.0000	0.9333	1.0000	1.0000	1.0000	1.0000	0.9889
Expedium	0.4615	0.6667	0.5385	0.4583	0.3077	0.6364	0.5115
Gezen	0.7692	0.8750	0.6667	0.8095	0.5556	1.0000	0.7793
NOVA	0.9167	1.0000	1.0000	0.8800	0.7857	1.0000	0.9304
Xia 3	0.8095	0.8947	0.8947	0.8049	0.7619	0.8500	0.8360
Macro‐average	0.8067	0.9020	0.8524	0.7898	0.6889	0.8894	0.8215
Micro‐average	0.8113	0.9057	0.8585	0.7972	0.7075	0.8868	0.8278
Sensitivity							
A‐Spine (EVO)	0.7222	0.9444	0.9444	0.8333	0.6667	1.0000	0.8518
Armstrong	0.8235	0.8235	0.7647	0.7647	0.6471	0.8824	0.7843
CDH	0.9375	0.8750	0.9375	0.9375	1.0000	0.8750	0.9271
Expedium	0.6667	0.8889	0.7778	0.6111	0.4444	0.7778	0.6945
Gezen	0.5882	0.8235	0.5882	0.5000	0.2941	0.7059	0.5833
NOVA	0.9167	1.0000	1.0000	0.9167	0.9167	0.9167	0.9445
Xia 3	1.0000	1.0000	1.0000	0.9706	0.9412	1.0000	0.9853
Macro‐average	0.8078	0.9079	0.8590	0.7906	0.7014	0.8797	0.8244
Micro‐average	0.8113	0.9057	0.8585	0.7972	0.7075	0.8868	0.8278
F1 score							
A‐Spine (EVO)	0.7879	0.9444	0.9714	0.8333	0.7273	0.9231	0.8646
Armstrong	0.8235	0.9032	0.8125	0.7536	0.6286	0.8824	0.8006
CDH	0.9677	0.9032	0.9677	0.9677	1.0000	0.9333	0.9566
Expedium	0.5455	0.7619	0.6364	0.5238	0.3636	0.7000	0.5885
Gezen	0.6667	0.8485	0.6250	0.6182	0.3846	0.8276	0.6618
NOVA	0.9167	1.0000	1.0000	0.8980	0.8462	0.9565	0.9362
Xia 3	0.8947	0.9444	0.9444	0.8800	0.8421	0.9189	0.9041
Macro‐average	0.8004	0.9008	0.8511	0.7821	0.6846	0.8774	0.8161
Micro‐average	0.8113	0.9057	0.8585	0.7972	0.7075	0.8868	0.8278
AUC							
A‐Spine (EVO)	0.9609	0.9968	0.9994	0.9634	0.9356	0.9968	0.9755
Armstrong	0.9445	0.9947	0.9689	0.9552	0.9359	0.9670	0.9610
CDH	0.9993	0.9972	0.9944	0.9995	1.0000	0.9979	0.9981
Expedium	0.9278	0.9851	0.9393	0.9439	0.8866	0.9805	0.9439
Gezen	0.8612	0.9451	0.9233	0.8946	0.8334	0.9299	0.8979
NOVA	0.9938	1.0000	1.0000	0.9949	0.9911	1.0000	0.9966
Xia 3	0.9782	0.9914	0.9934	0.9835	0.9828	0.9775	0.9845
Macro‐average	0.9560	0.9902	0.9772	0.9643	0.9420	0.9821	0.9686
Micro‐average	0.9542	0.9869	0.9809	0.9644	0.9463	0.9771	0.9683

Abbreviation: Concat, concatenated.

**TABLE 3:** Brand‐based evaluation of ensemble models, regardless of the presence of crosslinks.

Metrics	All	AP + LP	AP + Lat + Concat	AP + Lat + Merge	Lat + Concat	Average
Accuracy	0.9245	0.9434	0.9340	0.9340	0.9340	0.9340
Kappa score	0.9115	0.9335	0.9225	0.9224	0.9225	0.9225
Precision						
A‐Spine (EVO)	0.9474	0.9474	0.9474	0.9000	0.9474	0.9379
Armstrong	0.8889	0.9412	0.9375	0.9412	1.0000	0.9418
CDH	1.0000	1.0000	1.0000	1.0000	0.9375	0.9875
Expedium	0.7273	0.8000	0.8000	0.8000	0.8000	0.7855
Gezen	1.0000	1.0000	0.9333	1.0000	0.9333	0.9733
NOVA	1.0000	1.0000	1.0000	1.0000	1.0000	1.0000
Xia 3	0.8947	0.8947	0.8947	0.8947	0.8947	0.8947
Macro‐average	0.9226	0.9405	0.9304	0.9337	0.9304	0.9315
Micro‐average	0.9245	0.9434	0.9340	0.9340	0.9340	0.9340
Sensitivity						
A‐Spine (EVO)	1.0000	1.0000	1.0000	1.0000	1.0000	1.0000
Armstrong	0.9412	0.9412	0.8824	0.9412	0.8824	0.9177
CDH	0.9375	0.9375	0.9375	0.9375	0.9375	0.9375
Expedium	0.8889	0.8889	0.8889	0.8889	0.8889	0.8889
Gezen	0.7059	0.8235	0.8235	0.8235	0.8235	0.8000
NOVA	1.0000	1.0000	1.0000	0.9167	1.0000	0.9833
Xia 3	1.0000	1.0000	1.0000	1.0000	1.0000	1.0000
Macro‐average	0.9248	0.9416	0.9332	0.9297	0.9332	0.9325
Micro‐average	0.9245	0.9434	0.9340	0.9340	0.9340	0.9340
F1 score						
A‐Spine (EVO)	0.9730	0.9730	0.9730	0.9474	0.9730	0.9679
Armstrong	0.9143	0.9412	0.9091	0.9412	0.9375	0.9287
CDH	0.9677	0.9677	0.9677	0.9677	0.9375	0.9617
Expedium	0.8000	0.8421	0.8421	0.8421	0.8421	0.8337
Gezen	0.8276	0.9032	0.8750	0.9032	0.8750	0.8768
NOVA	1.0000	1.0000	1.0000	0.9565	1.0000	0.9913
Xia 3	0.9444	0.9444	0.9444	0.9444	0.9444	0.9444
Macro‐average	0.9181	0.9388	0.9302	0.9289	0.9299	0.9292
Micro‐average	0.9245	0.9434	0.9340	0.9340	0.9340	0.9340
AUC						
A‐Spine (EVO)	0.9981	0.9956	0.9987	0.9949	0.9994	0.9973
Armstrong	0.9828	0.9868	0.9874	0.9822	0.9881	0.9855
CDH	0.9993	0.9993	0.9986	1.0000	0.9965	0.9987
Expedium	0.9828	0.9828	0.9794	0.9874	0.9794	0.9824
Gezen	0.8876	0.9048	0.9035	0.8916	0.9161	0.9007
NOVA	1.0000	1.0000	1.0000	1.0000	1.0000	1.0000
Xia 3	0.9848	0.9861	0.9881	0.9835	0.9907	0.9866
Macro‐average	0.9811	0.9834	0.9842	0.9842	0.9854	0.9837
Micro‐average	0.9746	0.9830	0.9821	0.9748	0.9842	0.9797

Abbreviation: Concat, concatenated.

**TABLE 4:** Comparison between single and ensemble models, regardless of the presence of crosslinks.

Metrics	Accuracy	Precision‐macro	Sensitivity‐macro	F1 score‐macro
MAIA models				
AP model	0.8113	0.8067	0.8078	0.8004
Lat model	0.9057	0.902	0.9079	0.9008
Concat model	0.8585	0.8524	0.8590	0.8511
Merge model				
Dual images	0.7972	0.7898	0.7906	0.7821
AP images	0.7075	0.6889	0.7014	0.6846
Lat images	0.8868	0.8894	0.8797	0.8774
Average	0.8278	0.8215	0.8244	0.8161
Ensemble models				
All	0.9245	0.9226	0.9248	0.9181
AP + LP	0.9434	0.9405	0.9416	0.9388
AP + Lat + Concat	0.9340	0.9304	0.9332	0.9302
AP + Lat + Merge	0.9340	0.9337	0.9297	0.9289
Lat + Concat	0.9340	0.9304	0.9332	0.9299
Average	0.9340	0.9315	0.9325	0.9292

Abbreviations: Concat, concatenated; MAIA, Medical Artificial Intelligence Aggregator.

To investigate whether the presence of a crosslink influenced the performance of the DL models, we analyzed the performance of the model based on the PRS, with or without the crosslink. Both MAIA (Table S1) and ensemble models (Table S2) performed better when a crosslink was included.

**FIGURE 3:**
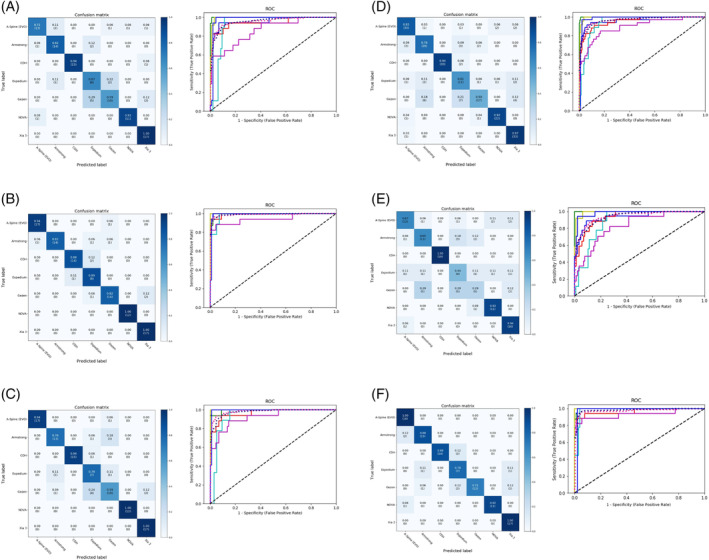
Confusion matrices (left) and receiver operating characteristic (ROC) curves (right) of MAIA models regardless of the presence of crosslinks. Range of the area under the ROC curve (AUC): (A) AP model, 0.93–1; (B) Lat model, 0.95–1; (C) Concat model, 0.92–1; (D) Merge model trained on dual images, 0.89–1; (E) Merge model trained on AP images, 0.83–1; and (F) Merge model trained on lateral images, 0.93–1. The *x*‐ and *y*‐axis in the confusion matrices represent the true labels and the predicted labels, respectively. Darker blue in the confusion matrices represents higher values. Lines are colored to indicate the following: blue, ROC curve of A‐Spine; red, ROC curve of Armstrong; green, ROC curve of CDH; light blue, ROC curve of Expedium; lavender, ROC curve of Gezen; yellow‐green, ROC curve of NOVA; dark blue, ROC curve of Xia 3; shocking pink dotted line, micro‐average ROC curve; oriental blue dotted line, macro‐average ROC curve.

**FIGURE 4:**
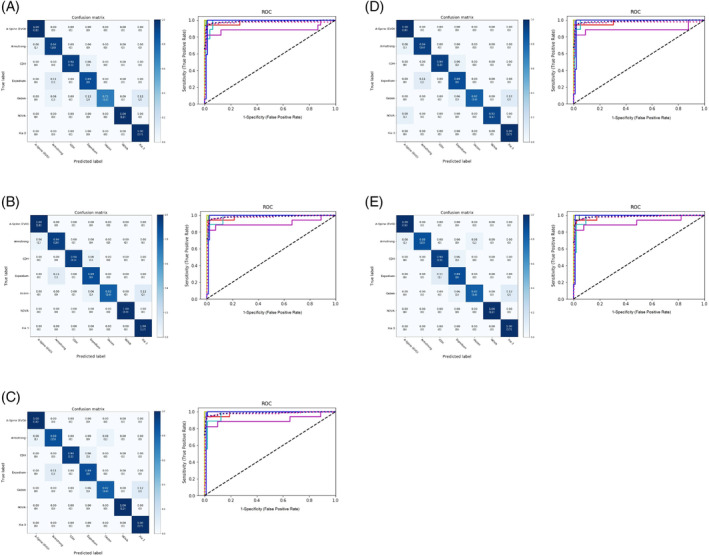
Confusion matrixes (left) and receiver operating characteristic (ROC) curves (right) of ensemble models regardless of the presence of crosslinks. (A) T Range of the area under the ROC curve (AUC): (A) All ensemble models, 0.89–1; (B) AP + Lat ensemble model, 0.9–1; (C) AP + Lat + Concat ensemble model, 0.9–1; (D) AP + Lat + Merge ensemble model, 0.97–1; (E) Lat + Concat ensemble model, 0.92–1. The *x*‐ and *y*‐axis in the confusion matrixes represent the true labels and the predicted labels, respectively. Darker blue in the confusion matrixes represents higher values. Lines are colored to indicate the following: blue, ROC curve of A‐Spine; red, ROC curve of Armstrong; green, ROC curve of CDH; light blue, ROC curve of Expedium; lavender, ROC curve of Gezen; yellow green, ROC curve of NOVA; dark blue, ROC curve of Xia 3; shocking pink dotted line, micro‐average ROC curve; oriental blue dotted line, macro‐average ROC curve.

**FIGURE 5:**
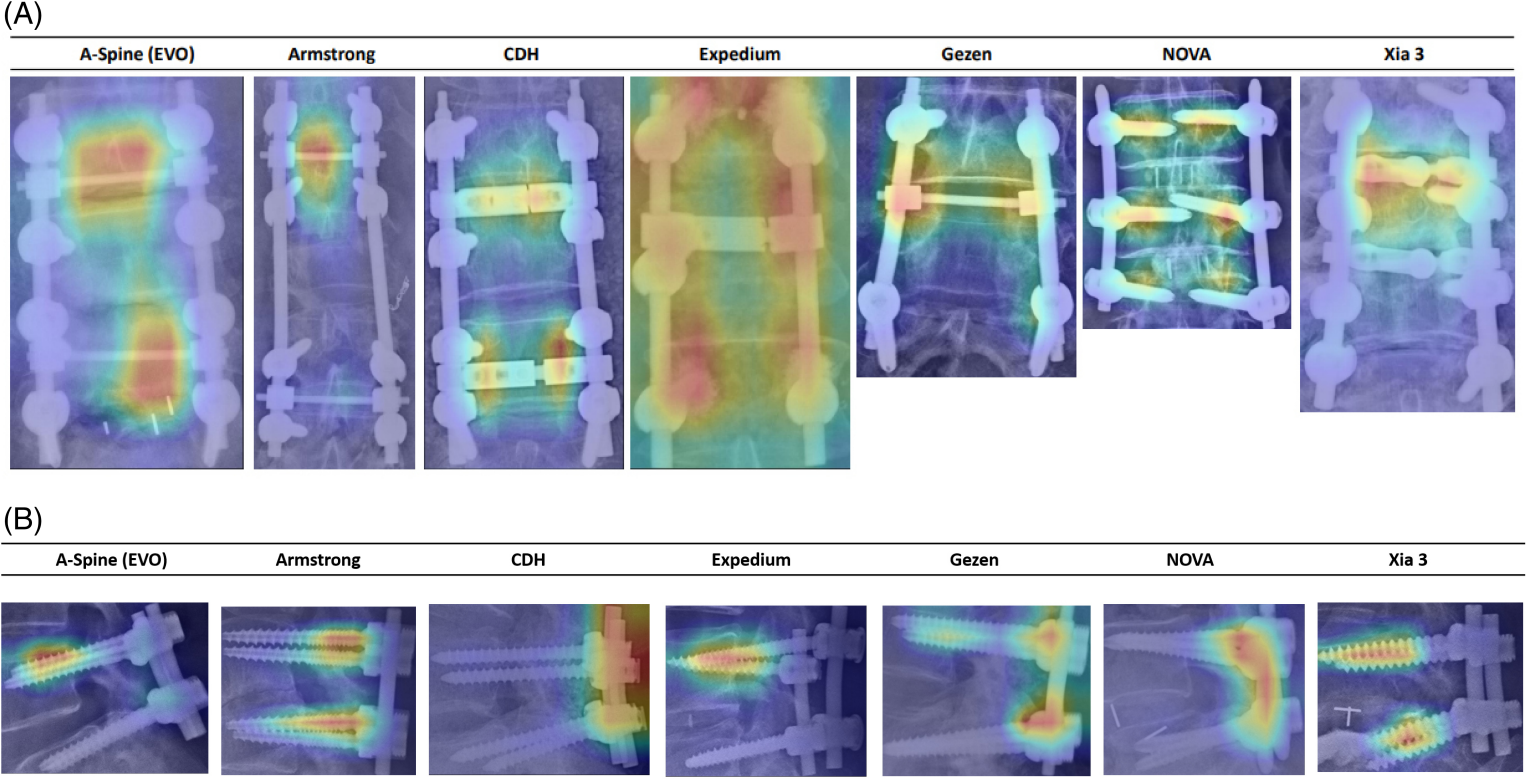
Illustration of gradient‐weighted class activation mapping (Grad‐CAM) on plain radiographs of spines of anteroposterior or lateral images to identify brands. (A) Heatmap on plain radiographs of spines on the anteroposterior image for seven brands of screws. No crosslink was used in the NOVA group because the screw was designed for the minimally invasive approach. (B) Heatmap on plain radiographs of spines in the lateral image for seven brands of screws.

Results of the analysis of the confusion matrix and ROC curve in the MAIA models, regardless of crosslink, are shown in Figure 3 and Figures S1 and S2. Results of the analysis of the confusion matrix and ROC curve in the ensemble models, regardless of crosslink, are shown in Figure 4 and Figures S3 and S4. To confirm the ability of the models to identify the features of the screws, we manually reviewed the Grad‐CAMs as evaluated by all models and reported by MAIA. The DL models focused on the discriminative regions of either screw pitch or crosslink to correctly classify PS‐based instruments (Figure 5, Figure S5).

In the performance comparison between human readers and the DL models, the accuracy among human readers ranged from 0.37 to 0.89 (Table 5). The least accurate performance (0.37) was that of a medical student. In contrast, the average accuracy of four attending orthopedic spine surgeons was 0.823 ± 0.047. Test completion required an average of 752 ± 263 s (range: 587–1250 s) for human readers and 3 s for all models. The ensemble models achieved an accuracy of 0.89–1.00. The performance of these ensemble models was not inferior to those of experienced orthopedic spine surgeons.

**TABLE 5:** Performance comparison between human readers and deep learning (DL) models (*n* = 27).

	Correct classification	Accuracy
Human reader		
Reader 1	22	0.81
Reader 2	24	0.89
Reader 3	21	0.78
Reader 4	24	0.89
Reader 5	21	0.78
Reader 6	10	0.37
Reader 7	21	0.78
Reader 8	22	0.81
Reader 9	16	0.59
Reader 10	20	0.74
Reader 11	17	0.63
MAIA models		
AP model	20	0.74
Lat model	27	1.00
Concat model	24	0.89
Merge model on AP images	19	0.70
Merge model on Lat images	26	0.96
Ensemble models		
All models	25	0.93
AP + Lat	27	1.00
AP + Lat + Concat	26	0.96
AP + Lat + Merge	26	0.96
Lat + Concat	27	1.00

*Note*: Readers 1–6 were invited from the present institution. Readers 7–11 were invited from another institution. Their specialties and levels are summarized below.

Reader 1: Orthopedic spine surgeon, attending surgeon.

Reader 2: Orthopedic spine surgeon, attending surgeon.

Reader 3: Orthopedic resident.

Reader 4: Orthopedic spine fellow.

Reader 5: Orthopedic surgeon specialized in arthroplasty, attending surgeon.

Reader 6: Medical student.

Reader 7: Orthopedic spine surgeon, attending surgeon.

Reader 8: Orthopedic spine surgeon, attending surgeon.

Reader 9: Orthopedic resident.

Reader 10: Orthopedic spine fellow.

Reader 11: Orthopedic surgeon specialized in limb trauma, attending surgeon.

Abbreviations: Concat, concatenated; MAIA, Medical Artificial Intelligence Aggregator.

Regarding external validation (*n* = 31), the accuracy of the Lat model was 0.8824 (Table S3), and the accuracy of the Lat + Concat ensemble model was 0.9412 (Table S4). The testing model for automated identification of PSs is available at https://140.136.158.62/web_VF/x-ray-ps.html.

## 4 DISCUSSION

In this study, we developed and validated DL models to identify PS‐based instrumentation. Our results revealed that using the lateral image as the ground truth resulted in a more stable performance by our DL models; using the ensemble method also improved results. The performance of the ensemble models was not inferior to that of experienced orthopedic spine surgeons. Taken together, these results suggest that these improved DL models can be an alternative means to identify PS‐based instrumentation on PRS in clinical practice.

A DL model has been used to identify 15 types of cervical plating systems with 85.8% accuracy in the top‐1 model based on 402 smartphone images.^11^ Another DL model is able to identify 9 types of cervical plating systems with an accuracy of 91.5% in the top‐1 model based on 321 PRS.^12^ The above‐mentioned studies used the same three brands of cervical plating systems (Medtronic Atlantis Vision, Depuy Synthes CSLP, and Depuy Synthes Skyline).^11^, ^12^ However, different ground truths were used; one was based on smartphone images,^11^ and the other was based on PRS.^12^ Consistently, the use of the top‐1 statistical method achieved good discriminative performance in this study. We believe that our use of YOLOv5 to crop out the screw trajectory before brand identification and conducting ensemble analysis underlies these results.

AP images of PRS were often used as ground truths in DL models for identifying the implant design in different anatomic locations such as the cervical spine, knee, and hips.^8^, ^11^ However, in the present study, we found that the ground truth of the lateral image provided a more stable result in single models. This phenomenon may in part result from the fact that different implants are designed for different types of anatomic fixation. For example, cervical plating is fixed at the anterior vertebrae, and the whole construct can be easily visualized on an AP image, as with knee and hip arthroplasties.^11^, ^12^ In PS‐based instrumentation, the trajectory is placed along the pedicle, and screw constructs at the neck and body may not be as clearly visible on AP images due to interference from the screw head and connecting rod.

Different PS systems may have distinct constructs (e.g., cylindrical vs. conical cores)^24^ or differences in pitch, tip, and crosslink. Theoretically, the entire PS construct can be easily visualized from the head to the distal tip on a lateral image. The crosslink could be clearly visualized on an AP image, as evidenced by the Grad‐CAMs heatmaps. The use of crosslinks also improved their performance. Several factors may be responsible for this observation. First, the crosslink was still partly visible on the lateral image because of the non‐parallel relationship between the beam of the X‐ray projector and the PS. Second, a crosslink is used to connect both sides of the PS‐based constructs, especially in two‐level and multi‐level fixations, in order to increase pullout strength. Thus, the performance of the lateral image‐based DL model increases with the number of screws that can be seen on the lateral image of the PRS.

In the present study, the screw body was red (very important) in all A‐Spine and Expedium heatmaps, while the screw head was red (very important) in all CDH and NOVA heatmaps. Pedicle screws from different manufacturers have their own characteristics and unique constructs, which may help to partially explain why the DL models judged different locations of PS from different manufacturers based on our ground truths. However, the mechanisms underlying the above phenomenon remain to be investigated. The pronounced red intensity in Expedium's heatmap may be in part because PS manufactured by Armstrong, CDH, and Gezen were frequently misjudged as Expedium by MAIA models and ensemble models due to similar constructs.

Clinical investigations have reported excellent accuracy of DL models in discriminating hip arthroplasties using different models, achieving 99.6%–100% accuracy.^8^, ^10^, ^25^ One study^11^ reported an accuracy of 94.4% in identifying 15 different cervical plating implants. Studies using DL models to identify hip arthroplasties achieved a ROC of 0.98^9^ to 0.99^8^, ^25^ discrimination, and accuracy reached 100%.^10^ One open‐access website, Implant Identifier,^26^ automatically identifies several arthroplasties of the hip, knee, elbow, shoulder, ankle, and wrist.^8^ However, this web application has not been used to identify spine implants such as cervical plating and PS‐based systems, despite the recent increase in the number of spine fusion surgeries performed.^27^, ^28^

The present study found comparable predictive performance between ensemble models and experienced orthopedic surgeons, which is in agreement with the result of a meta‐analysis.^29^ The potential of DL models as supplementary diagnostic tools to improve the diagnostic accuracy of clinicians has been demonstrated.^6^, ^30^, ^31^ With the assistance of a DL model, the incidence of misinterpretation of radiologic images reduces by 47.0%.^6^ DL models not only help to improve diagnostic accuracy but also speed up diagnosis, which is extremely important for emergency medicine clinicians.^6^, ^31^ Moreover, DL models as supplementary diagnostic tools may help clinicians with limited training in musculoskeletal imaging to enhance fracture detection accuracy.^30^ The current findings also suggest that the ensemble models may help inexperienced orthopedic surgeons to identify the brands of the existing implants.

While the performance of our models in identifying PS‐based instrumentations is encouraging, these results are limited to the identification of only seven implant types. Brand or manufacturer preferences vary in different countries and hospitals. An expansion of the models to identify other brands of PS‐based instrumentation is required to make them clinically useful.^28^ Accordingly, we expect to collect and externally validate data from a multi‐center study that expands the number of samples for each implant design analyzed to reach peak generalizability of the ground truth.^32^ Using MAIA software for model training and testing allows us to efficiently include new datasets and re‐train the models in an automated fashion. Moreover, we plan to make the models available on the smartphone, the method commonly used clinically to communicate medical images.^33^ Of the different methods used to identify the instrumentation brand preoperatively, the most reliable and efficient is to require preoperative registration via government or insurance policy.

## 5 CONCLUSION

The ensemble model achieved a more stable performance in identifying seven PS‐based screws commonly used in our clinics compared with any single model. The proposed ensemble models may serve as a supplementary diagnostic tool to help inexperienced orthopedic surgeons to correctly identify the brand of PS‐based instrumentation. Optimizing the generalizability of the ground truth by including more brands of implants from other healthcare systems will increase the clinical usefulness of the algorithm. The current results will facilitate a better preoperative planning for patients who need implant removal for revision surgery.

## CONFLICT OF INTEREST STATEMENT

The authors declare no conflicts of interest.

## Supporting information


Data S1.

## References

[1] Song M , Sun K , Li Z , et al. Stress distribution of different lumbar posterior pedicle screw insertion techniques: a combination study of finite element analysis and biomechanical test. Sci Rep. 2021;11(1):12968. doi:10.1038/s41598-021-90686-6 34155224 PMC8217271

[2] Ghiselli G , Wang JC , Bhatia NN , Hsu WK , Dawson EG . Adjacent segment degeneration in the lumbar spine. J Bone Joint Surg Am. 2004;86(7):1497‐1503. doi:10.2106/00004623-200407000-00020 15252099

[3] Fritsch EW , Heisel J , Rupp S . The failed back surgery syndrome: reasons, intraoperative findings, and long‐term results: a report of 182 operative treatments. Spine (Phila Pa 1976). 1996;21(5):626‐633. doi:10.1097/00007632-199603010-00017 8852320

[4] Cheng CT , Wang Y , Chen HW , et al. A scalable physician‐level deep learning algorithm detects universal trauma on pelvic radiographs. Nat Commun. 2021;12(1):1066. doi:10.1038/s41467-021-21311-3 33594071 PMC7887334

[5] Li YC , Chen HH , Horng‐Shing LH , Hondar Wu HT , Chang MC , Chou PH . Can a deep‐learning model for the automated detection of vertebral fractures approach the performance level of human subspecialists? Clin Orthop Relat Res. 2021;479(7):1598‐1612. doi:10.1097/corr.0000000000001685 33651768 PMC8208416

[6] Lindsey R , Daluiski A , Chopra S , et al. Deep neural network improves fracture detection by clinicians. Proc Natl Acad Sci USA. 2018;115(45):11591‐11596. doi:10.1073/pnas.1806905115 30348771 PMC6233134

[7] Chou PH , Jou TH , Wu HH , et al. Ground truth generalizability affects performance of the artificial intelligence model in automated vertebral fracture detection on plain lateral radiographs of the spine. Spine J. 2022;22(4):511‐523. doi:10.1016/j.spinee.2021.10.020 34737066

[8] Karnuta JM , Haeberle HS , Luu BC , et al. Artificial intelligence to identify arthroplasty implants from radiographs of the hip. J Arthroplast. 2021;36(7):S290‐S294.e291. doi:10.1016/j.arth.2020.11.015 33281020

[9] Klemt C , Uzosike AC , Cohen‐Levy WB , Harvey MJ , Subih MA , Kwon YM . The ability of deep learning models to identify total hip and knee arthroplasty implant design from plain radiographs. J Am Acad Orthop Surg. 2022;30(9):409‐415. doi:10.5435/jaaos-d-21-00771 35139038

[10] Borjali A , Chen AF , Muratoglu OK , Morid MA , Varadarajan KM . Detecting total hip replacement prosthesis design on plain radiographs using deep convolutional neural network. J Orthop Res. 2020;38(7):1465‐1471. doi:10.1002/jor.24617 31997411

[11] Schwartz JT , Valliani AA , Arvind V , et al. Identification of anterior cervical spinal instrumentation using a smartphone application powered by machine learning. Spine (Phila Pa 1976). 2022;47(9):E407‐E414. doi:10.1097/brs.0000000000004172 34269759

[12] Huang KT , Silva MA , See AP , et al. A computer vision approach to identifying the manufacturer and model of anterior cervical spinal hardware. J Neurosurg Spine. 2019;31:844‐850. doi:10.3171/2019.6.Spine19463 31491759

[13] Yang HS , Kim KR , Kim S , Park JY . Deep learning application in spinal implant identification. Spine (Phila Pa 1976). 2021;46(5):E318‐E324. doi:10.1097/BRS.0000000000003844 33534442

[14] Esfandiari H , Newell R , Anglin C , Street J , Hodgson AJ . A deep learning framework for segmentation and pose estimation of pedicle screw implants based on C‐arm fluoroscopy. Int J Comput Assist Radiol Surg. 2018;13(8):1269‐1282. doi:10.1007/s11548-018-1776-9 29808466

[15] Esfandiari H , Anglin C , Guy P , Hodgson AJ . A deep learning‐based approach for localization of pedicle regions in preoperative CT scans. CAOS 2018;2:46–50.

[16] Tan M , Le QV . EfficientNet: rethinking model scaling for convolutional neural networks. 2019. CoRR;abs/1905.11946.

[17] Deng J , Dong W , Socher R , Li L‐J , Li K , Fei‐Fei L . Imagenet: a large‐scale hierarchical image database; 2009. IEEE Conference on Computer Vision and Pattern Recognition; 2009.

[18] Perez L , Wang J . The effectiveness of data augmentation in image classification using deep learning. 2017; CoRR. abs/1712.04621.

[19] Kingma D , Ba J . Adam: a method for stochastic optimization. International Conference on Learning Representations; 2014.

[20] Smith LN . A disciplined approach to neural network hyper‐parameters: part 1–learning rate, batch size, momentum, and weight decay. *arXiv e‐Prints*. 2018:arXiv:1803.09820.

[21] Huang G , Li Y , Pleiss G , Liu Z , Hopcroft JE , Weinberger KQ . Snapshot ensembles: train 1, get M for free. 2017. CoRR;abs/1704.00109.

[22] Selvaraju RR , Cogswell M , Das A , Vedantam R , Parikh D , Batra D . Grad‐CAM: visual explanations from deep networks via gradient‐based localization. Paper Presented at: 2017 IEEE International Conference on Computer Vision (ICCV); 22–29 Oct. 2017 2017.

[23] Research randomizer. Accessed July 1, 2020. http://www.randomizer.org

[24] Shea TM , Laun J , Gonzalez‐Blohm SA , et al. Designs and techniques that improve the pullout strength of pedicle screws in osteoporotic vertebrae: current status. Biomed Res Int. 2014;2014:748393. doi:10.1155/2014/748393 24724097 PMC3958762

[25] Kang YJ , Yoo JI , Cha YH , Park CH , Kim JT . Machine learning‐based identification of hip arthroplasty designs. J Orthop Translat. 2020;21:13‐17. doi:10.1016/j.jot.2019.11.004 32071870 PMC7013104

[26] Desai P . Implant identifier. Accessed July 1, 2020. https://implantidentifier.app/

[27] Petscavage‐Thomas J , Ouyang T , Bible J . Spine fixation hardware: an update. AJR Am J Roentgenol. 2020;215(3):534‐544. doi:10.2214/ajr.20.22810 32755228

[28] Sheikh SR , Thompson NR , Benzel E , et al. Can we justify it? Trends in the utilization of spinal fusions and associated reimbursement. Neurosurgery. 2020;86(2):E193‐e202. doi:10.1093/neuros/nyz400 31574148

[29] Kuo RYL , Harrison C , Curran TA , et al. Artificial intelligence in fracture detection: a systematic review and meta‐analysis. Radiology. 2022;304(1):50‐62. doi:10.1148/radiol.211785 35348381 PMC9270679

[30] Anderson PG , Baum GL , Keathley N , et al. Deep learning assistance closes the accuracy gap in fracture detection across clinician types. Clin Orthop Relat Res. 2023;481(3):580‐588. doi:10.1097/corr.0000000000002385 36083847 PMC9928835

[31] Fu T , Viswanathan V , Attia A , et al. Assessing the potential of a deep learning tool to improve fracture detection by radiologists and emergency physicians on extremity radiographs. Acad Radiol. 2024;31(5):1989‐1999. doi:10.1016/j.acra.2023.10.042 37993303

[32] Titano JJ , Badgeley M , Schefflein J , et al. Automated deep‐neural‐network surveillance of cranial images for acute neurologic events. Nat Med. 2018;24(9):1337‐1341. doi:10.1038/s41591-018-0147-y 30104767

[33] Bullard TB , Rosenberg MS , Ladde J , Razack N , Villalobos HJ , Papa L . Digital images taken with a mobile phone can assist in the triage of neurosurgical patients to a level 1 trauma centre. J Telemed Telecare. 2013;19(2):80‐83. doi:10.1177/1357633x13476228 23528786

