# Supplementary material for: Development and validation of deep learning models for identifying the brand of pedicle screws on plain spine radiographs
Source: JOR Spine. 2024 Sep 17;7(3):e70001. doi: 10.1002/jsp2.70001 (PMC11406509; doi:10.1002/jsp2.70001)
Supplement: Supplementary file 1 — Data S1. [file JSP2-7-e70001-s001.docx]

**Supplementary Figure S1.** Confusion matrixes for identification of instrumentation with crosslink in MAIA models: (a) AP model; (b) Lat model; (c) Concat model; (d) Merge model trained on dual images; (e) Merge model trained on AP images; (f) Merge model trained on Lat images.


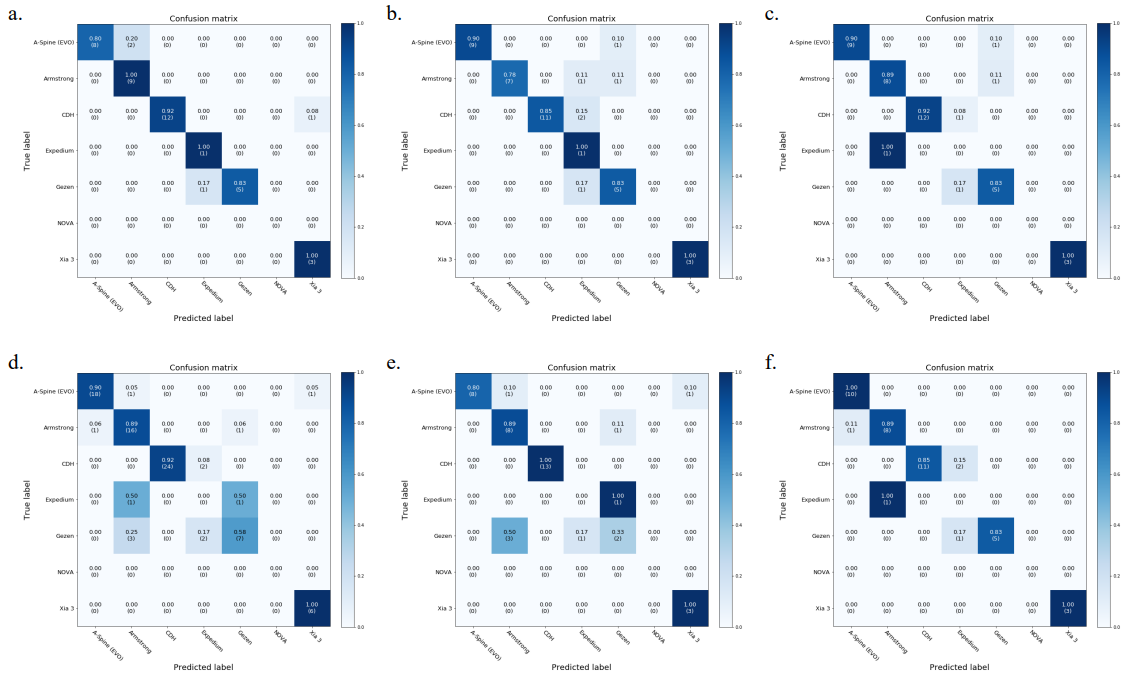
The x- and y-axis represent true labels and predicted labels, respectively. Darker blue represents higher values.

**Supplementary Figure S2.** Confusion matrixes for identification of instrumentation without cross link in MAIA models: (a) AP model; (b) Lat model; (c) Concat model; (d) Merge model trained on dual images; (e) Merge model trained on AP images; (f) Merge model trained on Lat images.


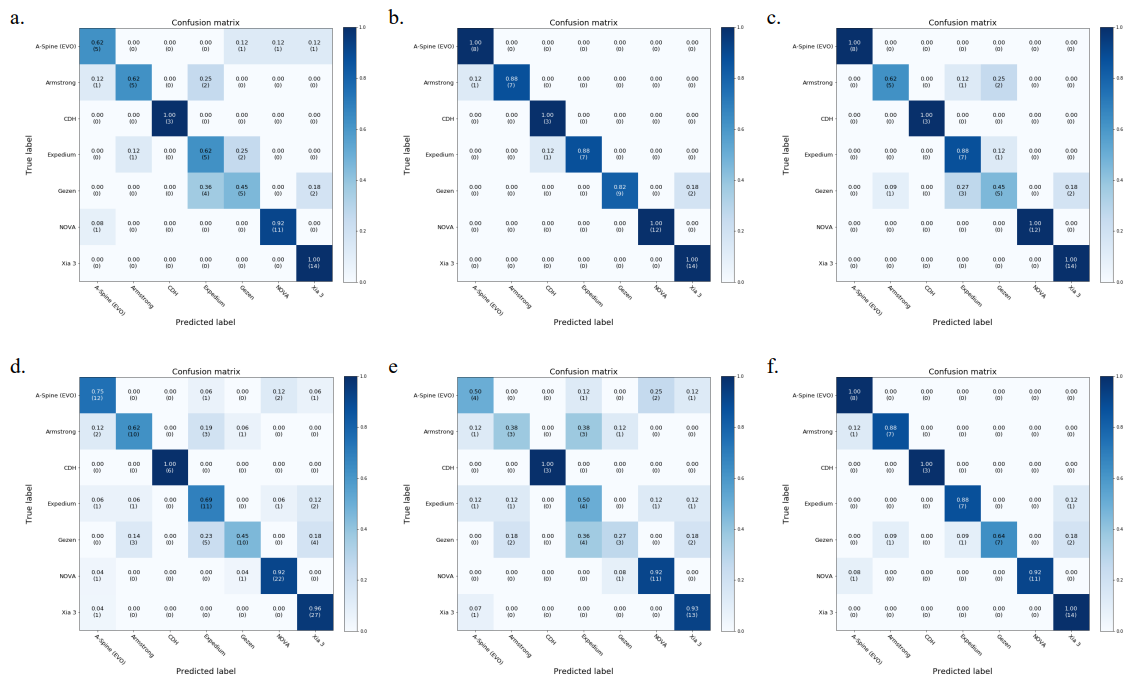
The x- and y-axis represent true labels and predicted labels, respectively. Darker blue represents higher values.

**Supplementary Figure S3.** Confusion matrixes for identification of instrumentation with crosslink in the ensemble models: (a) All; (b) AP + Lat ; (c) AP + Lat + Concat model; (d) AP + Lat + Merge model; (e) Lat + Concat model.

The x- and y-axis represent true labels and predicted labels, respectively. Darker blue represents higher values.
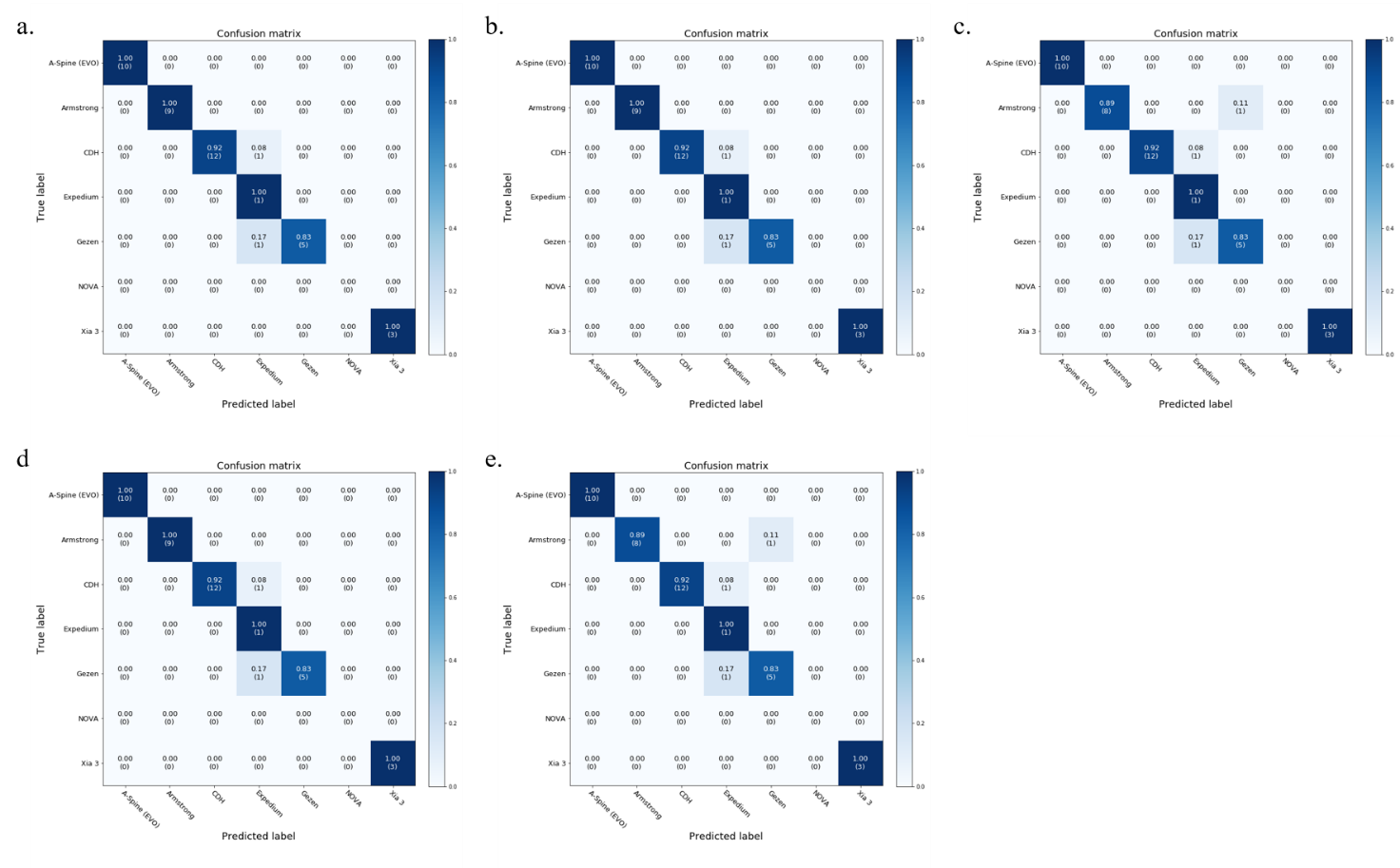


**Supplementary Figure S4.** Confusion matrixes for identification of instrumentation without crosslink in the ensemble models: (a) All; (b) AP + Lat; (c) AP + Lat + Concat model; (d) AP + Lat + Merge model, (e) Lat + Concat model.

The x- and y-axis represent true labels and predicted labels, respectively. Darker blue represents higher values.
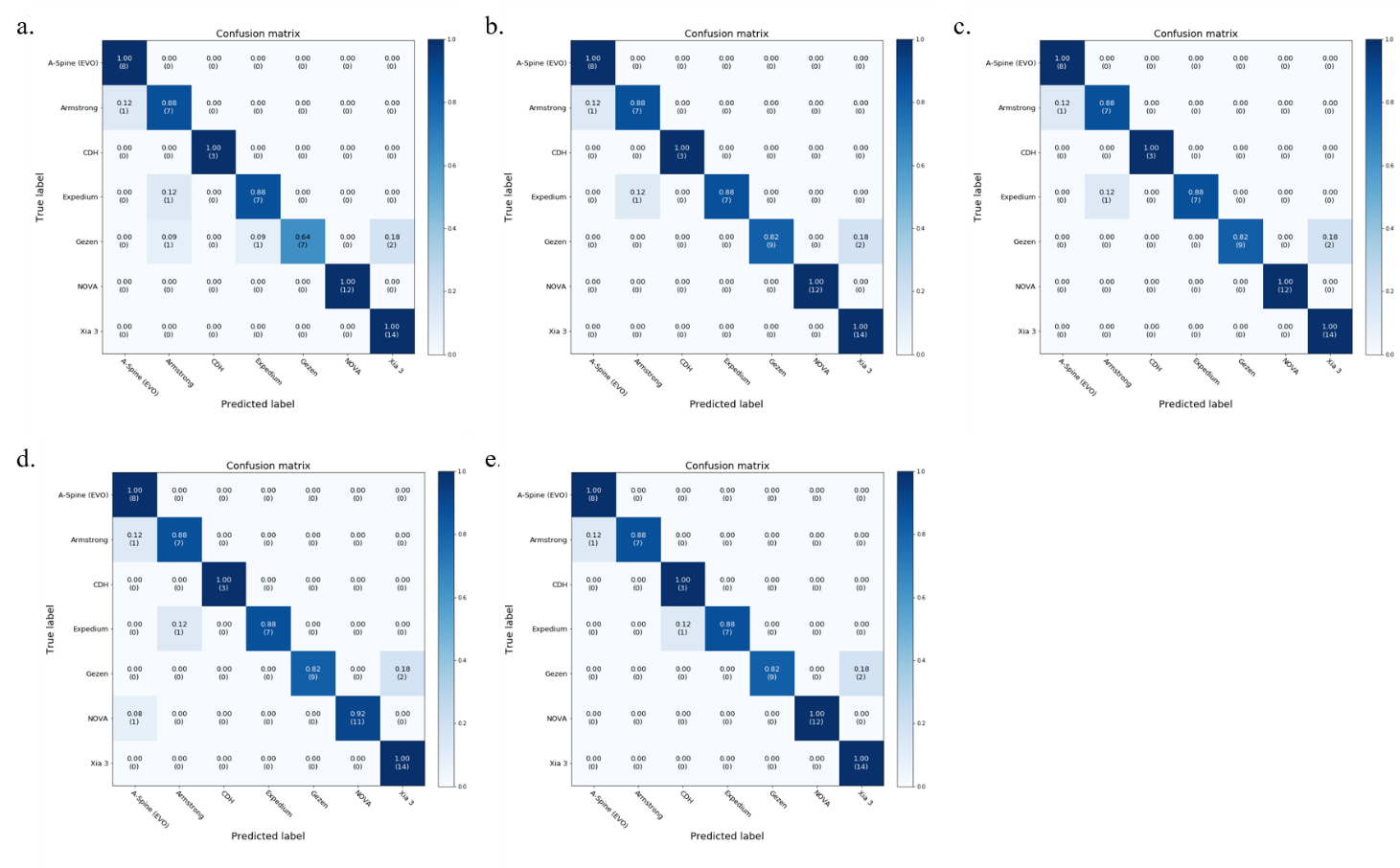


**Supplementary Figure S5.** Illustration of the Concatenated model in identifying pedicle screw-based instrumentations in Gradient-weighted Class Activation Mapping (Grad-CAMs) heat maps: (a) A-spine (EVO); (b) Armstrong; (c) CDH; (d) Expedium; (e) Gezen; (f) NOVA; (g) Xia 3.


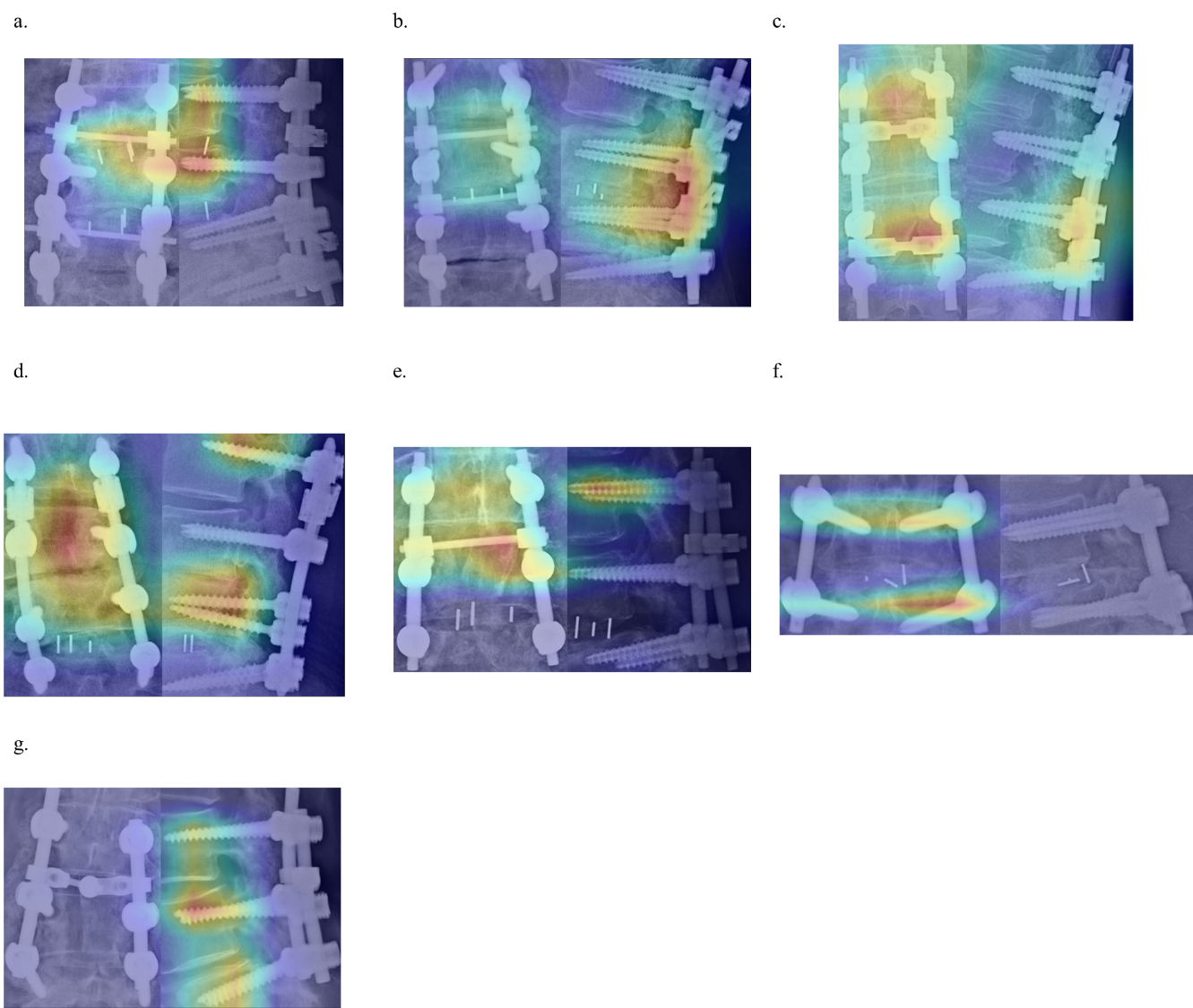


**Supplementary Table S1.** Accuracy, precision, sensitivity, F1-score, and AUC in MAIA models based on plain radiographs of spines with or without crosslink**.**

| **Metrics** | AP model | | Lat model | | Concat model | | Merge model | | | | | | Average | |
| --- | --- | --- | --- | --- | --- | --- | --- | --- | --- | --- | --- | --- | --- | --- |
|  |  |  |  |  |  |  | Dual  images | | AP  images | | Lat images | |  |  |
|  | CL | no CL | CL | no CL | CL | no CL | CL | no CL | CL | no CL | CL | no CL | CL | no CL |
| **Accuracy** | 0.9048 | 0.7500 | 0.8571 | 0.9375 | 0.8810 | 0.8438 | 0.8452 | 0.7656 | 0.8095 | 0.6406 | 0.8810 | 0.8906 | 0.8631 | 0.8047 |
| **Kappa Score** | 0.8787 | 0.7010 | 0.8215 | 0.9254 | 0.8483 | 0.8135 | 0.8020 | 0.7200 | 0.7547 | 0.5703 | 0.8487 | 0.8695 | 0.8257 | 0.7666 |
| **Precision** |  |  |  |  |  |  |  |  |  |  |  |  |  |  |
| A-Spine (EVO) | 1.0000 | 0.7143 | 1.0000 | 0.8889 | 1.0000 | 1.0000 | 0.9474 | 0.7059 | 1.0000 | 0.5714 | 0.9091 | 0.8000 | 0.9761 | 0.7801 |
| Armstrong | 0.8182 | 0.8333 | 1.0000 | 1.0000 | 0.8889 | 0.8333 | 0.7619 | 0.7143 | 0.6667 | 0.5000 | 0.8889 | 0.8750 | 0.8374 | 0.7927 |
| CDH | 1.0000 | 1.0000 | 1.0000 | 0.7500 | 1.0000 | 1.0000 | 1.0000 | 1.0000 | 1.0000 | 1.0000 | 1.0000 | 1.0000 | 1.0000 | 0.9583 |
| Expedium | 0.5000 | 0.4545 | 0.2000 | 1.0000 | 0.0000 | 0.6364 | 0.0000 | 0.5500 | 0.0000 | 0.3333 | 0.0000 | 0.8750 | 0.1167 | 0.6415 |
| Gezen | 1.0000 | 0.6250 | 0.7143 | 1.0000 | 0.7143 | 0.6250 | 0.7778 | 0.8333 | 0.5000 | 0.6000 | 1.0000 | 1.0000 | 0.7844 | 0.7806 |
| NOVA | - | 0.9167 | - | 1.0000 | - | 1.0000 | - | 0.8800 | - | 0.7857 | - | 1.0000 | - | 0.9304 |
| Xia 3 | 0.7500 | 0.8235 | 1.0000 | 0.8750 | 1.0000 | 0.8750 | 0.8571 | 0.7941 | 0.7500 | 0.7647 | 1.0000 | 0.8235 | 0.8929 | 0.8260 |
| Macro-avg | 0.8447 | 0.7668 | 0.8190 | 0.9306 | 0.7672 | 0.8528 | 0.7240 | 0.7825 | 0.6528 | 0.6507 | 0.7997 | 0.9105 | 0.7679 | 0.8157 |
| Micro-avg | 0.9048 | 0.7500 | 0.8571 | 0.9375 | 0.8810 | 0.8438 | 0.8452 | 0.7656 | 0.8095 | 0.6406 | 0.8810 | 0.8906 | 0.8631 | 0.8047 |
| **Sensitivity** |  |  |  |  |  |  |  |  |  |  |  |  |  |  |
| A-Spine (EVO) | 0.8000 | 0.6250 | 0.9000 | 1.0000 | 0.9000 | 1.0000 | 0.9000 | 0.7500 | 0.8000 | 0.5000 | 1.0000 | 1.0000 | 0.8833 | 0.8125 |
| Armstrong | 1.0000 | 0.6250 | 0.7778 | 0.8750 | 0.8889 | 0.6250 | 0.8889 | 0.6250 | 0.8889 | 0.3750 | 0.8889 | 0.8750 | 0.8889 | 0.6667 |
| CDH | 0.9231 | 1.0000 | 0.8462 | 1.0000 | 0.9231 | 1.0000 | 0.9231 | 1.0000 | 1.0000 | 1.0000 | 0.8462 | 1.0000 | 0.9103 | 1.0000 |
| Expedium | 1.0000 | 0.6250 | 1.0000 | 0.8750 | 0.0000 | 0.8750 | 0.0000 | 0.6875 | 0.0000 | 0.5000 | 0.0000 | 0.8750 | 0.3333 | 0.7396 |
| Gezen | 0.8333 | 0.4545 | 0.8333 | 0.8182 | 0.8333 | 0.4545 | 0.5833 | 0.4545 | 0.3333 | 0.2727 | 0.8333 | 0.6364 | 0.7083 | 0.5151 |
| NOVA | - | 0.9167 | - | 1.0000 | - | 1.0000 | - | 0.9167 | - | 0.9167 | - | 0.9167 | - | 0.9445 |
| Xia 3 | 1.0000 | 1.0000 | 1.0000 | 1.0000 | 1.0000 | 1.0000 | 1.0000 | 0.9643 | 1.0000 | 0.9286 | 1.0000 | 1.0000 | 1.0000 | 0.9822 |
| Macro-avg | 0.9261 | 0.7495 | 0.8929 | 0.9383 | 0.7575 | 0.8506 | 0.7159 | 0.7711 | 0.6704 | 0.6419 | 0.7614 | 0.9004 | 0.7874 | 0.8086 |
| Micro-avg | 0.9048 | 0.7500 | 0.8571 | 0.9375 | 0.8810 | 0.8438 | 0.8452 | 0.7656 | 0.8095 | 0.6406 | 0.8810 | 0.8906 | 0.8631 | 0.8047 |
| **F1 score** |  |  |  |  |  |  |  |  |  |  |  |  |  |  |
| A-Spine (EVO) | 0.8889 | 0.6667 | 0.9474 | 0.9412 | 0.9474 | 1.0000 | 0.9231 | 0.7273 | 0.8889 | 0.5333 | 0.9524 | 0.8889 | 0.9247 | 0.7929 |
| Armstrong | 0.9000 | 0.7143 | 0.8750 | 0.9333 | 0.8889 | 0.7143 | 0.8205 | 0.6667 | 0.7619 | 0.4286 | 0.8889 | 0.8750 | 0.8559 | 0.7220 |
| CDH | 0.9600 | 1.0000 | 0.9167 | 0.8571 | 0.9600 | 1.0000 | 0.9600 | 1.0000 | 1.0000 | 1.0000 | 0.9167 | 1.0000 | 0.9522 | 0.9762 |
| Expedium | 0.6667 | 0.5263 | 0.3333 | 0.9333 | 0.0000 | 0.7368 | 0.0000 | 0.6111 | 0.0000 | 0.4000 | 0.0000 | 0.8750 | 0.1667 | 0.6804 |
| Gezen | 0.9091 | 0.5263 | 0.7692 | 0.9000 | 0.7692 | 0.5263 | 0.6667 | 0.5882 | 0.4000 | 0.3750 | 0.9091 | 0.7778 | 0.7372 | 0.6156 |
| NOVA | - | 0.9167 | - | 1.0000 | - | 1.0000 | - | 0.8980 | - | 0.8462 | - | 0.9565 | - | 0.9362 |
| Xia 3 | 0.8571 | 0.9032 | 1.0000 | 0.9333 | 1.0000 | 0.9333 | 0.9231 | 0.8710 | 0.8571 | 0.8387 | 1.0000 | 0.9032 | 0.9396 | 0.8971 |
| Macro-avg | 0.8636 | 0.7505 | 0.8069 | 0.9283 | 0.7609 | 0.8444 | 0.7156 | 0.7660 | 0.6513 | 0.6317 | 0.7778 | 0.8966 | 0.7627 | 0.8029 |
| Micro-avg | 0.9048 | 0.7500 | 0.8571 | 0.9375 | 0.8810 | 0.8438 | 0.8452 | 0.7656 | 0.8095 | 0.6406 | 0.8810 | 0.8906 | 0.8631 | 0.8047 |
| **AUC** |  |  |  |  |  |  |  |  |  |  |  |  |  |  |
| A-Spine (EVO) | 0.9875 | 0.9263 | 0.9969 | 0.9955 | 1.0000 | 1.0000 | 0.9961 | 0.9157 | 0.9938 | 0.8482 | 1.0000 | 0.9911 | 0.9957 | 0.9461 |
| Armstrong | 1.0000 | 0.8839 | 1.0000 | 0.9844 | 0.9933 | 0.9353 | 0.9798 | 0.9230 | 0.9663 | 0.9085 | 0.9899 | 0.9420 | 0.9882 | 0.9295 |
| CDH | 0.9973 | 1.0000 | 1.0000 | 1.0000 | 0.9947 | 1.0000 | 0.9993 | 1.0000 | 1.0000 | 1.0000 | 0.9973 | 1.0000 | 0.9981 | 1.0000 |
| Expedium | 0.9756 | 0.8929 | 1.0000 | 0.9955 | 0.9268 | 0.9397 | 0.9329 | 0.9392 | 0.9512 | 0.8393 | 0.9268 | 0.9911 | 0.9522 | 0.9330 |
| Gezen | 0.9954 | 0.7547 | 0.9907 | 0.9245 | 0.9815 | 0.8782 | 0.9595 | 0.8443 | 0.9259 | 0.7547 | 0.9815 | 0.8816 | 0.9724 | 0.8397 |
| NOVA | - | 0.9888 | - | 1.0000 | - | 1.0000 | - | 0.9908 | - | 0.9840 | - | 1.0000 | _ | 0.9939 |
| Xia 3 | 1.0000 | 0.9614 | 1.0000 | 0.9871 | 1.0000 | 0.9886 | 1.0000 | 0.9696 | 1.0000 | 0.9671 | 1.0000 | 0.9600 | 1.0000 | 0.9723 |
| Macro-avg | 0.9926 | 0.9231 | 0.9979 | 0.9882 | 0.9827 | 0.9688 | 0.9779 | 0.9443 | 0.9729 | 0.9076 | 0.9826 | 0.9734 | 0.9844 | 0.9509 |
| Micro-avg | 0.9940 | 0.9220 | 0.9958 | 0.9811 | 0.9925 | 0.9723 | 0.9904 | 0.9429 | 0.9868 | 0.9080 | 0.9938 | 0.9648 | 0.9922 | 0.9485 |

CL, crosslink; Macro-avg, macro-average; Micro-avg, micro-average

**Supplementary Table S2.** Accuracy, precision, sensitivity, F1-score, and AUC in ensemble models based on plain radiographs of spines with or without crosslink.

| **Metrics** | **All** | | **AP + Lat** | | **AP + Lat + Concat** | | **AP + Lat + Merge** | | **Lat + Concat** | | **Average** | |
| --- | --- | --- | --- | --- | --- | --- | --- | --- | --- | --- | --- | --- |
|  | CL | no CL | CL | no CL | CL | no CL | CL | no CL | CL | no CL | CL | no CL |
| **Accuracy** | 0.9524 | 0.9063 | 0.9524 | 0.9375 | 0.9286 | 0.9375 | 0.9524 | 0.9219 | 0.9286 | 0.9375 | 0.9429 | 0.9281 |
| **Kappa Score** | 0.9394 | 0.8882 | 0.9394 | 0.9253 | 0.9092 | 0.9253 | 0.9394 | 0.9068 | 0.9092 | 0.9254 | 0.9273 | 0.9142 |
| **Precision** | | | | | | | | | | | | |
| A-Spine (EVO) | 1.0000 | 0.8889 | 1.0000 | 0.8889 | 1.0000 | 0.8889 | 1.0000 | 0.8000 | 1.0000 | 0.8889 | 1.0000 | 0.8711 |
| Armstrong | 1.0000 | 0.7778 | 1.0000 | 0.8750 | 1.0000 | 0.8750 | 1.0000 | 0.8750 | 1.0000 | 1.0000 | 1.0000 | 0.8806 |
| CDH | 1.0000 | 1.0000 | 1.0000 | 1.0000 | 1.0000 | 1.0000 | 1.0000 | 1.0000 | 1.0000 | 0.7500 | 1.0000 | 0.9500 |
| Expedium | 0.3333 | 0.8750 | 0.3333 | 1.0000 | 0.3333 | 1.0000 | 0.3333 | 1.0000 | 0.3333 | 1.0000 | 0.3333 | 0.9750 |
| Gezen | 1.0000 | 1.0000 | 1.0000 | 1.0000 | 0.8333 | 1.0000 | 1.0000 | 1.0000 | 0.8333 | 1.0000 | 0.9333 | 1.0000 |
| NOVA | - | 1.0000 | - | 1.0000 | - | 1.0000 | - | 1.0000 | - | 1.0000 | - | 1.0000 |
| Xia 3 | 1.0000 | 0.8750 | 1.0000 | 0.8750 | 1.0000 | 0.8750 | 1.0000 | 0.8750 | 1.0000 | 0.8750 | 1.0000 | 0.8750 |
| Macro-average | 0.8889 | 0.9167 | 0.8889 | 0.9484 | 0.8611 | 0.9484 | 0.8889 | 0.9357 | 0.8611 | 0.9306 | 0.8778 | 0.9360 |
| Micro-average | 0.9524 | 0.9063 | 0.9524 | 0.9375 | 0.9286 | 0.9375 | 0.9524 | 0.9219 | 0.9286 | 0.9375 | 0.9429 | 0.9281 |
| **Sensitivity** | | | | | | | | | | | | |
| A-Spine (EVO) | 1.0000 | 1.0000 | 1.0000 | 1.0000 | 1.0000 | 1.0000 | 1.0000 | 1.0000 | 1.0000 | 1.0000 | 1.0000 | 1.0000 |
| Armstrong | 1.0000 | 0.8750 | 1.0000 | 0.8750 | 0.8889 | 0.8750 | 1.0000 | 0.8750 | 0.8889 | 0.8750 | 0.9556 | 0.8750 |
| CDH | 0.9231 | 1.0000 | 0.9231 | 1.0000 | 0.9231 | 1.0000 | 0.9231 | 1.0000 | 0.9231 | 1.0000 | 0.9231 | 1.0000 |
| Expedium | 1.0000 | 0.8750 | 1.0000 | 0.8750 | 1.0000 | 0.8750 | 1.0000 | 0.8750 | 1.0000 | 0.8750 | 1.0000 | 0.8750 |
| Gezen | 0.8333 | 0.6364 | 0.8333 | 0.8182 | 0.8333 | 0.8182 | 0.8333 | 0.8182 | 0.8333 | 0.8182 | 0.8333 | 0.7818 |
| NOVA | - | 1.0000 | - | 1.0000 | - | 1.0000 | - | 0.9167 | - | 1.0000 | - | 0.9833 |
| Xia 3 | 1.0000 | 1.0000 | 1.0000 | 1.0000 | 1.0000 | 1.0000 | 1.0000 | 1.0000 | 1.0000 | 1.0000 | 1.0000 | 1.0000 |
| Macro-average | 0.9594 | 0.9123 | 0.9594 | 0.9383 | 0.9409 | 0.9383 | 0.9594 | 0.9264 | 0.9409 | 0.9383 | 0.9520 | 0.9307 |
| Micro-average | 0.9524 | 0.9063 | 0.9524 | 0.9375 | 0.9286 | 0.9375 | 0.9524 | 0.9219 | 0.9286 | 0.9375 | 0.9429 | 0.9281 |
| **F1 score** | | | | | | | | | | | | |
| A-Spine (EVO) | 1.0000 | 0.9412 | 1.0000 | 0.9412 | 1.0000 | 0.9412 | 1.0000 | 0.8889 | 1.0000 | 0.9412 | 1.0000 | 0.9307 |
| Armstrong | 1.0000 | 0.8235 | 1.0000 | 0.8750 | 0.9412 | 0.8750 | 1.0000 | 0.8750 | 0.9412 | 0.9333 | 0.9765 | 0.8764 |
| CDH | 0.9600 | 1.0000 | 0.9600 | 1.0000 | 0.9600 | 1.0000 | 0.9600 | 1.0000 | 0.9600 | 0.8571 | 0.9600 | 0.9714 |
| Expedium | 0.5000 | 0.8750 | 0.5000 | 0.9333 | 0.5000 | 0.9333 | 0.5000 | 0.9333 | 0.5000 | 0.9333 | 0.5000 | 0.9216 |
| Gezen | 0.9091 | 0.7778 | 0.9091 | 0.9000 | 0.8333 | 0.9000 | 0.9091 | 0.9000 | 0.8333 | 0.9000 | 0.8788 | 0.8756 |
| NOVA | - | 1.0000 | - | 1.0000 | - | 1.0000 | - | 0.9565 | - | 1.0000 | - | 0.9913 |
| Xia 3 | 1.0000 | 0.9333 | 1.0000 | 0.9333 | 1.0000 | 0.9333 | 1.0000 | 0.9333 | 1.0000 | 0.9333 | 1.0000 | 0.9333 |
| Macro-average | 0.8948 | 0.9073 | 0.8948 | 0.9404 | 0.8724 | 0.9404 | 0.8948 | 0.9267 | 0.8724 | 0.9283 | 0.8858 | 0.9286 |
| Micro-average | 0.9524 | 0.9063 | 0.9524 | 0.9375 | 0.9286 | 0.9375 | 0.9524 | 0.9219 | 0.9286 | 0.9375 | 0.9429 | 0.9281 |
| **AUC** | | | | | | | | | | | | |
| A-Spine (EVO) | 1.0000 | 0.9955 | 1.0000 | 0.9911 | 1.0000 | 1.0000 | 1.0000 | 0.9866 | 1.0000 | 1.0000 | 1.0000 | 0.9946 |
| Armstrong | 1.0000 | 0.9665 | 1.0000 | 0.9710 | 1.0000 | 0.9732 | 1.0000 | 0.9621 | 1.0000 | 0.9732 | 1.0000 | 0.9692 |
| CDH | 1.0000 | 1.0000 | 1.0000 | 1.0000 | 1.0000 | 1.0000 | 1.0000 | 1.0000 | 0.9947 | 1.0000 | 0.9989 | 1.0000 |
| Expedium | 0.9512 | 0.9911 | 1.0000 | 0.9844 | 0.9512 | 0.9844 | 0.9756 | 0.9933 | 0.9512 | 0.9911 | 0.9658 | 0.9889 |
| Gezen | 0.9815 | 0.8456 | 0.9907 | 0.8456 | 0.9861 | 0.8491 | 0.9861 | 0.8456 | 0.9861 | 0.8662 | 0.9861 | 0.8504 |
| NOVA | - | 1.0000 | - | 1.0000 | - | 1.0000 | - | 1.0000 | - | 1.0000 | - | 1.0000 |
| Xia 3 | 1.0000 | 0.9714 | 1.0000 | 0.9757 | 1.0000 | 0.9786 | 1.0000 | 0.9686 | 1.0000 | 0.9843 | 1.0000 | 0.9757 |
| Macro-average | - | 0.9742 | - | 0.9747 | - | 0.9760 | - | 0.9765 | - | 0.9806 | - | 0.9764 |
| Micro-average | 0.9965 | 0.9591 | 0.9984 | 0.9689 | 0.9965 | 0.9697 | 0.9974 | 0.9587 | 0.9954 | 0.9748 | 0.9968 | 0.9662 |

CL, crosslink; Lat, lateral; Concat, concatenated

**Supplementary Table S3.** Performance of external validation of MAIA models, regardless of presence of crosslink.

| **Metrics** | **AP model** | **Lat model** | **Concat model** | **Merged model** | | | **Average** |
| --- | --- | --- | --- | --- | --- | --- | --- |
|  |  |  |  | **Dual images** | **AP images** | **Lat images** |  |
| **Accuracy** | 0.5294 | 0.8824 | 0.8824 | 0.6471 | 0.4412 | 0.8529 | 0.7059 |
| **Kappa Score** | 0.4409 | 0.8609 | 0.8591 | 0.5757 | 0.3299 | 0.8227 | 0.6482 |
| **Precision** |  |  |  |  |  |  |  |
| A-Spine (EVO) | 0.6667 | 1.0000 | 1.0000 | 0.6000 | 0.5000 | 0.6667 | 0.7389 |
| Armstrong | 0.5714 | 1.0000 | 0.9000 | 0.6500 | 0.4444 | 0.8182 | 0.7307 |
| CDH | 1.0000 | 1.0000 | 1.0000 | 0.7500 | 0.5000 | 1.0000 | 0.8750 |
| Expedium | 0.5000 | 1.0000 | 0.7500 | 0.8000 | 0.5000 | 1.0000 | 0.7583 |
| Gezen | 0.6000 | 0.8000 | 0.8000 | 0.8333 | 0.6667 | 1.0000 | 0.7833 |
| NOVA | 0.4167 | 0.7143 | 1.0000 | 0.5294 | 0.3636 | 0.8333 | 0.6429 |
| Xia 3 | 0.3333 | 0.7500 | 0.7500 | 0.6667 | 0.3333 | 1.0000 | 0.6389 |
| Macro-average | 0.5840 | 0.8949 | 0.8857 | 0.6899 | 0.4726 | 0.9026 | 0.7383 |
| Micro-average | 0.5294 | 0.8824 | 0.8824 | 0.6471 | 0.4412 | 0.8529 | 0.7059 |
| **Sensitivity** |  |  |  |  |  |  |  |
| A-Spine (EVO) | 0.5000 | 1.0000 | 0.7500 | 0.7500 | 0.5000 | 1.0000 | 0.7500 |
| Armstrong | 0.4444 | 0.7778 | 1.0000 | 0.7222 | 0.4444 | 1.0000 | 0.7315 |
| CDH | 0.5000 | 0.7500 | 0.7500 | 0.3750 | 0.2500 | 0.5000 | 0.5208 |
| Expedium | 0.2500 | 1.0000 | 0.7500 | 0.5000 | 0.2500 | 0.7500 | 0.5833 |
| Gezen | 0.7500 | 1.0000 | 1.0000 | 0.6250 | 0.5000 | 0.7500 | 0.7708 |
| NOVA | 1.0000 | 1.0000 | 1.0000 | 0.9000 | 0.8000 | 1.0000 | 0.9500 |
| Xia 3 | 0.2500 | 0.7500 | 0.7500 | 0.5000 | 0.2500 | 0.7500 | 0.5417 |
| Macro-average | 0.5278 | 0.8968 | 0.8571 | 0.6246 | 0.4278 | 0.8214 | 0.6926 |
| Micro-average | 0.5294 | 0.8824 | 0.8824 | 0.6471 | 0.4412 | 0.8529 | 0.7059 |
| **F1 score** |  |  |  |  |  |  |  |
| A-Spine (EVO) | 0.5714 | 1.0000 | 0.8571 | 0.6667 | 0.5000 | 0.8000 | 0.7325 |
| Armstrong | 0.5000 | 0.8750 | 0.9474 | 0.6842 | 0.4444 | 0.9000 | 0.7252 |
| CDH | 0.6667 | 0.8571 | 0.8571 | 0.5000 | 0.3333 | 0.6667 | 0.6468 |
| Expedium | 0.3333 | 1.0000 | 0.7500 | 0.6154 | 0.3333 | 0.8571 | 0.6482 |
| Gezen | 0.6667 | 0.8889 | 0.8889 | 0.7143 | 0.5714 | 0.8571 | 0.7646 |
| NOVA | 0.5882 | 0.8333 | 1.0000 | 0.6667 | 0.5000 | 0.9091 | 0.7496 |
| Xia 3 | 0.2857 | 0.7500 | 0.7500 | 0.5714 | 0.2857 | 0.8571 | 0.5833 |
| Macro-average | 0.5160 | 0.8863 | 0.8644 | 0.6312 | 0.4240 | 0.8353 | 0.6929 |
| Micro-average | 0.5294 | 0.8824 | 0.8824 | 0.6471 | 0.4412 | 0.8529 | 0.7059 |
| **AUC** |  |  |  |  |  |  |  |
| A-Spine (EVO) | 0.8250 | 1.0000 | 1.0000 | 0.8938 | 0.8000 | 0.9833 | 0.9170 |
| Armstrong | 0.5822 | 0.9911 | 0.9956 | 0.8133 | 0.6356 | 0.9956 | 0.8356 |
| CDH | 1.0000 | 0.9917 | 1.0000 | 0.9417 | 0.9583 | 0.9250 | 0.9694 |
| Expedium | 0.7583 | 1.0000 | 0.9417 | 0.8833 | 0.7750 | 0.9917 | 0.8917 |
| Gezen | 0.9417 | 1.0000 | 1.0000 | 0.9729 | 0.9167 | 1.0000 | 0.9719 |
| NOVA | 0.9448 | 0.9862 | 1.0000 | 0.9517 | 0.9034 | 1.0000 | 0.9644 |
| Xia 3 | 0.7250 | 0.9917 | 0.7917 | 0.8792 | 0.7583 | 0.9833 | 0.8549 |
| Macro-average | 0.8426 | 0.9961 | 0.9761 | 0.9147 | 0.8361 | 0.9885 | 0.9257 |
| Micro-average | 0.7938 | 0.9905 | 0.9645 | 0.8835 | 0.7780 | 0.9797 | 0.8983 |

**Radiographic technique:** The radiography machine used a high-voltage generator (KONICA MINOLTA, Inc.) with a voltage of 80 and 96 KVP and an average current of 36 and 20 mAs for 71 and 51 mS for AP and lateral images, respectively.

Lat, lateral; Concat, concatenated

**Supplementary Table S4.** Performance of external validation of ensemble models, regardless of presence of crosslink.

| **Metrics** | **All** | **AP + LP** | **AP + Lat +**  **Concat** | **AP + Lat +**  **Merge** | **Lat +**  **Concat** | **Average** |
| --- | --- | --- | --- | --- | --- | --- |
| **Accuracy** | 0.8529 | 0.8529 | 0.9118 | 0.8235 | 0.9412 | 0.8765 |
| **Kappa Score** | 0.8249 | 0.8265 | 0.8946 | 0.7899 | 0.9298 | 0.8531 |
| **Precision** | | | | | | |
| A-Spine (EVO) | 1.0000 | 1.0000 | 1.0000 | 1.0000 | 1.0000 | 1.0000 |
| Armstrong | 0.8750 | 1.0000 | 0.8889 | 0.8750 | 1.0000 | 0.9278 |
| CDH | 1.0000 | 1.0000 | 1.0000 | 1.0000 | 1.0000 | 1.0000 |
| Expedium | 1.0000 | 1.0000 | 1.0000 | 1.0000 | 1.0000 | 1.0000 |
| Gezen | 1.0000 | 1.0000 | 1.0000 | 1.0000 | 1.0000 | 1.0000 |
| NOVA | 0.5556 | 0.5000 | 0.7143 | 0.5556 | 0.8333 | 0.6318 |
| Xia 3 | 1.0000 | 1.0000 | 1.0000 | 0.7500 | 0.7500 | 0.9000 |
| Macro-average | 0.9187 | 0.9286 | 0.9433 | 0.8829 | 0.9405 | 0.9228 |
| Micro-average | 0.8529 | 0.8529 | 0.9118 | 0.8235 | 0.9412 | 0.8765 |
| **Sensitivity** | | | | | | |
| A-Spine (EVO) | 1.0000 | 1.0000 | 1.0000 | 1.0000 | 1.0000 | 1.0000 |
| Armstrong | 0.8750 | 1.0000 | 0.8889 | 0.7778 | 1.0000 | 0.9083 |
| CDH | 1.0000 | 1.0000 | 1.0000 | 0.5000 | 1.0000 | 0.9000 |
| Expedium | 1.0000 | 1.0000 | 1.0000 | 0.7500 | 1.0000 | 0.9500 |
| Gezen | 1.0000 | 1.0000 | 1.0000 | 1.0000 | 1.0000 | 1.0000 |
| NOVA | 0.5556 | 0.5000 | 0.7143 | 1.0000 | 0.8333 | 0.7206 |
| Xia 3 | 1.0000 | 1.0000 | 1.0000 | 0.7500 | 0.7500 | 0.9000 |
| Macro-average | 0.9187 | 0.9286 | 0.9433 | 0.8254 | 0.9405 | 0.9113 |
| Micro-average | 0.8529 | 0.8529 | 0.9118 | 0.8235 | 0.9412 | 0.8765 |
| **F1 score** | | | | | | |
| A-Spine (EVO) | 1.0000 | 1.0000 | 1.0000 | 1.0000 | 1.0000 | 1.0000 |
| Armstrong | 0.8235 | 0.8000 | 0.8889 | 0.8235 | 1.0000 | 0.8672 |
| CDH | 0.8571 | 0.8571 | 1.0000 | 0.6667 | 0.8571 | 0.8476 |
| Expedium | 0.8571 | 1.0000 | 0.8571 | 0.8571 | 1.0000 | 0.9143 |
| Gezen | 1.0000 | 1.0000 | 1.0000 | 1.0000 | 1.0000 | 1.0000 |
| NOVA | 0.7143 | 0.6667 | 0.8333 | 0.7143 | 0.9091 | 0.7675 |
| Xia 3 | 0.8571 | 0.8571 | 0.8571 | 0.7500 | 0.7500 | 0.8143 |
| Macro-average | 0.8727 | 0.8830 | 0.9195 | 0.8302 | 0.9309 | 0.8873 |
| Micro-average | 0.8529 | 0.8529 | 0.9118 | 0.8235 | 0.9412 | 0.8765 |
| **AUC** | | | | | | |
| A-Spine (EVO) | 1.0000 | 1.0000 | 1.0000 | 1.0000 | 1.0000 | 1.0000 |
| Armstrong | 0.9911 | 0.9689 | 0.9956 | 0.9778 | 1.0000 | 0.9867 |
| CDH | 0.9917 | 1.0000 | 1.0000 | 0.9917 | 1.0000 | 0.9967 |
| Expedium | 1.0000 | 1.0000 | 1.0000 | 1.0000 | 1.0000 | 1.0000 |
| Gezen | 1.0000 | 1.0000 | 1.0000 | 1.0000 | 1.0000 | 1.0000 |
| NOVA | 1.0000 | 0.9793 | 1.0000 | 1.0000 | 1.0000 | 0.9959 |
| Xia 3 | 0.9250 | 0.9417 | 0.9500 | 0.9250 | 0.9750 | 0.9433 |
| Macro-average | 0.9922 | 0.9882 | 0.9954 | 0.9894 | 0.9982 | 0.9927 |
| Micro-average | 0.9854 | 0.9779 | 0.9889 | 0.9774 | 0.9960 | 0.9851 |

**Radiographic technique:** The radiography machine used a high-voltage generator (KONICA MINOLTA, Inc.) with a voltage of 80 and 96 KVP and an average current of 36 and 20 mAs for 71 and 51 mS for AP and lateral images, respectively.

Lat, lateral; Concat, concatenated
